# Health-promoting factors in higher education for a sustainable working life – protocol for a multicenter longitudinal study

**DOI:** 10.1186/s12889-020-8181-3

**Published:** 2020-02-14

**Authors:** U. Lindmark, I. Ahlstrand, A. Ekman, L. Berg, L. Hedén, J. Källstrand, M. Larsson, H. Nunstedt, L. Oxelmark, S. Pennbrant, A. Sundler, I. Larsson, Impact within the Swedish framework for “Health Research in Collaboration”

**Affiliations:** 1 Centre for Oral Health, Department of Natural Science and Biomedicine, School of Health and Welfare, Jönköping University, Jönköping, Sweden; 2 Department of Rehabilitation, School of Health and Welfare, Jönköping University, Jönköping, Sweden; 3 Department of Social Work, School of Health and Welfare, Jönköping University, Jönköping, Sweden; 4 Institute of Health and Care Sciences, the Sahlgrenska Academy, University of Gothenburg, Gothenburg, Sweden; 5 Faculty of Caring Science, Work Life, and Social Welfare, University of Borås, Borås, Sweden; 6 School of Health and Welfare, Halmstad University, Halmstad, Sweden; 7 School of Health and Education, University of Skövde, Skövde, Sweden; 8 Department of Health Sciences, University West, Trollhättan, Sweden

**Keywords:** Health promotion, Salutogenesis, Students’ health, Sustainable working life

## Abstract

**Background:**

The World Health Organization has highlighted the importance of health promotion for health service providers in order to ensure sustainable working life for individuals involved in providing health services. Such sustainability begins when students are preparing to manage their own future health and welfare in working life. It has been suggested that universities, employees and trainee health professionals should adopt or follow a salutogenic approach that not only complements the providing of information on known health risks but also favors health promotion strategies. This paper describes the study design and data collection methods in a planned study aiming to explore health-promoting factors for a sustainable working life among students in higher education within healthcare and social work.

**Methods:**

This protocol describes a multicenter longitudinal study involving Swedish students on higher education programs in the healthcare and social work sectors. In 2018, the study invited students on seven education programs at six universities to participate. These programs were for qualification as: biomedical laboratory scientists (*n* = 121); dental hygienists (*n* = 87); nurses (*n* = 1411); occupational therapists (*n* = 111); physiotherapists (*n* = 48); radiographers (*n* = 60); and, social workers (*n* = 443). In total, 2283 students were invited to participate. Participants completed a baseline, a self-reported questionnaire including six validated instruments measuring health-promoting factors and processes. There are to be five follow-up questionnaires. Three while the students are studying, one a year after graduating, and one three years after graduating. Each questionnaire captures different health-promoting dimensions, namely: health-promoting resources (i.e. sense of coherence); occupational balance; emotional intelligence; health and welfare; social interaction; and work and workplace experiences/perceptions.

**Discussion:**

This study focuses on the vastly important aspect of promoting a sustainable working life for healthcare and social work employees. In contrast to previous studies in this area, the present study uses different, validated instruments in health promotion, taking a salutogenic approach. It is hoped that, by stimulating the implementation of new strategies, the study’s findings will lead to education programs that prepare students better for a sustainable working life in healthcare and social work.

## Background

A sustainable workforce of professionals providing health and welfare services for individuals is a public health matter of national and global magnitude [55]. In Sweden, short and long-term absences on sick leave are high among employees in healthcare and social services [23]. Demands on health service professionals are severe and some even choose to leave their professions, even within the first years [1, 44]. The WHO has highlighted the importance of health promotion for health service providers in order to ensure a sustainable working life. Challenges related to health and sustainable working life are targeted by the Swedish Public Health Authorities, and research into health promotion and sustainable working life has been called for [1].

Education lays the foundation for a sustainable working life [29, 30, 47]. To manage challenges in their future working lives as providers of services for individuals, health and welfare students need to be prepared and properly equipped during their education programs [1]. Such preparation can be via either an “ill health approach” or a “health promotion approach”. Ill health during education has been studied, [12]. A longitudinal analysis of nursing education (LANE) study followed 4314 nursing students throughout their educational programs and the process of becoming registered nurses. Research in this study focused on: physical and psychological health; the drop-out rate among nursing students; and the first years of these students’ working lives [41]. The results showed a high incidence of neck, shoulder and/or back pain among nursing students and among newly graduated nurses [25]. In the transition between student life and working life, newly graduated nurses reported that sleep quality declined. This decline started from their last semester of nursing education and continued for 3 years into working life [15]. Other noteworthy findings have included: burnout symptoms on the program were an indicator of lower professional preparedness [39]; and the risk of burnout was one of the reasons nurses considered leaving the profession during their first 5 years [40]. Thus, in order to prevent stress-related ill health, an intervention was designed aimed at reducing issue-avoidance behavior and increasing engagement in proactive behavior among newly registered nurses [12].

In addition to focusing on and preventing ill health, health-promoting factors improve people’s capacity to develop abilities and resources to feel good and cope with different situations in a healthy way and are, by extension, essential for health and sustainable working life [29, 30]. Research has shown that: integration of a salutogenic (health promotion) approach in health education curricula is successful [9, 29], and that research is necessary to identify health-promoting factors during education programs [29, 30]. Health promotion takes place in settings as described in the Lifespan-setting-based framework [54]. University can be viewed as an important stage in the lifespan setting, and also a starting point in the transition into working life.

In explaining the concept of salutogenesis, Antonovsky [3] saw health as relative and as a continuum. He claimed that the most important research question centered on what causes health (salutogenesis) and not on what causes disease (pathogenesis) – [3, 19]. In a salutogenic approach, the focus is on understanding health-promoting factors and resources that maintain and improve progress toward health [18]. Health-promoting factors for a sustainable working life are related to a person’s lifestyle and health behavior (e.g. diet, physical activity, and smoking); the individual’s experience/perception of working conditions; and, the individual’s personal resources and abilities [24, 33]. Several health-promoting resources are important for people working in the healthcare and social services professions in the health and welfare sectors [6, 33]. Salutogenesis has been described as an asset for health and wellbeing. Today, salutogenesis is an umbrella theory covering many salutogenic elements and dimensions. Sense of coherence (SOC) is one of its concepts [20]. Salutogenic factors are essential for promoting health as well as sustainable working life.

A systematic review revealed a relationship between health and SOC [11]. A person with a high SOC characteristically has access to both personal and external resources, and the ability to use these resources in a health-promoting manner. As a result of this, life is experienced as comprehensible, manageable, and meaningful [2]. Thus, SOC seems to be: a health-promoting resource that strengthens the individual’s health [11], and a health-promoting factor in both an educational setting [27] and working life [17, 28, 49]. Salutogenic health-promoting factors in the field of healthcare work can be explained by the three “elements” of SOC, namely, comprehensibility, manageability, and meaningfulness. Comprehensibility covers both individual and group-related reflective skills. Examples include open-mindedness and a comprehensive view of the organization in question. Manageability includes attitudes, flexibility, and responsibility in the work situation. Meaningfulness relates to “rewards as reinforcements”; examples include happiness, satisfaction, confirmation, and belonging to a team [6, 33]. There have been studies of other health-promoting factors that are important for sustainable working life. Such factors include occupational balance, emotional intelligence, and social interaction/teamwork. Satisfaction with everyday activities (work and leisure) and the feeling of having occupational balance [50] are also important for wellbeing, health [13, 51, 52], and contentment [14]. It has also been shown that emotional intelligence is important in clinical work in healthcare [37, 42]. Similarly, collaboration [43, 45] in interdisciplinary teamwork among health professionals (social interaction not being overlooked in this) has been put forward as promoting a sustainable working life [48].

It has been suggested that universities, employees, and trainee health professionals should adopt or follow a salutogenic approach that not only complements the provision of information on known health risks but also favors health promotion strategies. When considering the possible implications of a salutogenic approach in health and social welfare related to higher education and work, the exploration of salutogenic factors and processes is valuable [31]. In this connection, to support sustainable working life, it is important to emphasize and develop salutogenic resources while students are undertaking their education programs [4, 26]. Thus, the study presented in this study protocol focuses on the impact of implementing, in education programs for the healthcare and social service professions, a salutogenic perspective on health-promoting factors for sustainable working life, starting during professional education.

The current paper describes the study design and data collection methods in this planned study.

### Aim

The overall aim is to explore health-promoting factors creating a sustainable working life for students on higher education programs in healthcare and social work.

### Objectives

The objectives of this study are to:

- Examine the distribution of possible health-promoting factors among the participants, and to determine whether these factors are associated with sustainability during the first 3 years of working life
- Explore similarities and differences in health-promoting factors among students within and between education programs.
- Explore similarities and differences in health-promoting factors among students concerning sociodemographic data.

### Methods and design

The current study is a multicenter longitudinal study involving Swedish students on higher education programs in the healthcare and social work sectors. The first data collection step took place in 2018. This provides a baseline for a larger longitudinal project covering the students’ education programs (i.e. 3 to 3.5 years) and the post graduation period as newly qualified and practicing professionals. In total, two follow-up assessments are planned during the education programs and two assessments after graduation.

The project explores salutogenic factors during higher education and the first years working as a professional. The data for the study is provided by questions regarding demographic characteristics and questionnaires regarding health promotion. The questionnaires cover salutogenic factors, the individual’s health, personal resources, and health behavior. This is a multicenter study within the Swedish framework for “Health Research in Collaboration”, involving six universities in southern Sweden. At each of these universities, two researchers are responsible for the study process. One of these researchers acts as a project manager.

### Participants

Those invited to participate were all students who, in the spring or fall of 2018, started one of the following healthcare or social work programs for qualification as: biomedical laboratory scientists (*n* = 146); dental hygienists (*n* = 86); nurses (*n* = 1392); occupational therapists (*n* = 110); physiotherapists (*n* = 48); radiographers (*n* = 60); and, social workers (*n* = 444). In this total population study, 2286 students were asked to participate. The only exclusion criterion was if students did not speak/read Swedish. The selection of participating programs was made by convenience i.e. focusing on the health and welfare sector and universities included in the Swedish collaborating framework for “Health Research in Collaboration”.

### Data collection

The survey is performed using a self-reported, web-based questionnaire (esMaker NX3 software). As the nursing and social work programs have two admissions each year and the other programs only have an admission in the fall, two baseline data collections were performed. Initially, each university administrative department provided lists of all the students’ email addresses. As these lists comprised all students who were still on their accepted programs 3 weeks after startup, students who had changed their study choice (e.g. other education programs or universities) at an early stage were avoided. To make it possible to follow the participants after program completion, they were requested to supply an email address via which they could subsequently receive questionnaires. Information about the study and the participation invitation were distributed (with the questionnaire) via the institutions’ (faculty, department, etc.) email systems. Initially, most of the students were approached by the researchers, who gave a short oral presentation about the aim and design of the study, and then gave the students the opportunity to ask questions.

Students were informed that, during their education programs, data collection would take place three times, i.e. the first (baseline), the fourth, and the last semester. The first questionnaire was distributed in spring/fall 2018. Three reminders were sent to those who did not respond to this questionnaire. The students who participated in 2018 will be included in a second data collection in fall 2019/spring 2020. This will be repeated for the third collection in fall 2020/spring 2021.

**Fig. 1:**
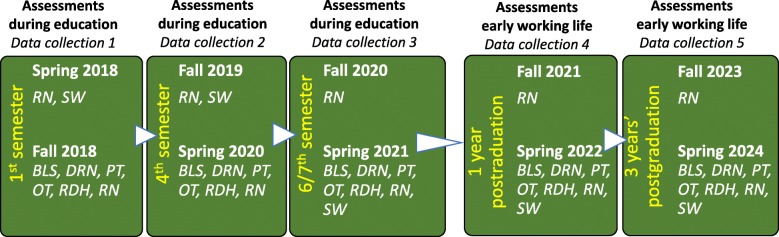
Assessments and students participating from the following from programmes: Biomedical Laboratory Scientists (BLS), Registered Nurses (RN), Occupational Therapists (OT), Physiotherapists (PT), Social Workers (SW), Diagnostic Radiology Nurses (DRN), Registered Dental Hygienists (RDH) (*N* = 2282 in 2018)

As regards assessments during early working life, data collection will take place one and 3 years after graduation. The students who participated during the education program will be invited to participate in this step. Figure 1 shows the data collection timeline.

#### Instruments

The baseline, web-based questionnaire covers demographic questions (gender, age, ethnicity, family situation, and residential area) and the reason for choosing the selected program and career (13 questions). Ten questions assess general health, welfare, and health behavior (i.e. diet, alcohol, tobacco use, sleep, and physical activity). These 10 questions and the demographic questions come from the Swedish Public Health survey [46], which is a large validated study on health, lifestyle, and living conditions conducted annually since 2004. Six validated instruments were chosen to measure health-promoting factors and processes (105 questions). The following instruments were used and are listed in Table 1: The life-oriented, 13-item Sense of Coherence (SOC) Scale [2], the Occupational Balance Questionnaire (OBQ, 11 items) [53], the Trait Emotional Intelligence Questionnaires – Short Form (TEIQue-SF, 30 items) [35], the Salutogenic Health Indicator Scale (SHIS, 12 items) [5]; and QBS Nordic (five items) [8], which measures social interaction/teamwork. After student graduation, the questionnaires are to be modified to suit a workplace setting. The “Work Experience Measurement Scale” (32 items) [34] will also be added.

**Table 1:** Descriptions of the instruments used in the longitudinal study

Instrument	Items	References	Captures	Dimensions
Sense of coherence scale (SOC-13)	13	[2]	Health promoting resources	Comprehensibility, manageability, meaningfulness
Occupational Balance Questionnaire (OBQ)	11	[53]	Occupational balance	Occupational areas, occupations with different characteristics, time use
Trait Emotional Intelligence Questionnaire-Short Form (TEIQue-SF)	30	[35, 36]	Emotional intelligence	Emotional coping ability
Salutogenic Health Indicator Scale (SHIS)	12	[5]	Health and welfare	Intrapersonal characteristics (IPC) and interactive function (IAF)
The General Nordic Questionnaire (QPS Nordic)	5	[8]	Social interactions	Psychological and social aspects
Work Experience Measurement Scale (WEMS)	32	[34]	Experience/perception of work and workplace	Supportive working conditions, individual inner experiences, self-determination, perception of time, leadership and change initiatives

All instruments were chosen with regard to the salutogenic framework and research in the area. The usefulness of the included instruments has been considered useful and appropriate for both the period of the education programs and the period after graduation. Several of the instruments have been used within health occupational research and with groups of students. All researchers have significant and complementary experience in the areas of healthcare and social welfare as well as in health promotion and occupational research. The questionnaire was produced and discussed in close collaboration with reference groups that included students, student unions (health sections) and staff at student healthcare centers. Before the final version of the questionnaire was distributed, a pilot study was conducted with a representative student sample (*n* = 50). Minor corrections were made concerning the layout of the survey, but there were no comments concerning the questions included.

##### Sense of coherence

In the study, health-promoting resources are measured using sense of coherence (SOC) and the SOC-13 “life questionnaire” [2]. SOC examines the individual’s ability to adopt healthy choices and behaviors. The instrument comprises 13 items (questions/statements). It has five items related to comprehensibility, four related to manageability and four related to meaningfulness. There is a seven-point semantic scale for responding to each item. The responses attract a total of 13–91 points, a high score indicating a strong SOC. An example of a comprehensibility-related item is as follows: “When you talk to people, do you have a feeling that they do not understand you?” The scale here runs from “Never have this feeling” to “Always have this feeling”. The following is an example of a manageability-related item: “When you do something that gives you a good feeling” (participants have to select a response/continuation indicating their “optimism”). The scale here runs from “It is certain that I will go on feeling good” to “It is certain that something will happen to spoil the feeling”. The following is an example of a meaningfulness-related item: “Doing the things I do every day is” (participants have to select a response/continuation indicating their “feelings”). The scale here runs from “A source of deep pleasure and satisfaction” to “A source of pain and boredom” ([2], p. 190). The scale was translated into Swedish by Langius and Bjorvell [16] and has been proved to be psychometrically sound. Previous studies have shown SOC to be: a good indicator of health, extremely valid, and, reliable [10].

##### Occupational balance

Occupational balance is measured by the Occupational Balance Questionnaire (OBQ) [53]. The OBQ focuses on satisfaction with the amount, and variation of occupations. It comprises 11 items that are responded to via a four-point ordinal scale ranging from “completely disagree” (scored at 0) to “completely agree” (scored at 3). High scores mean high levels of experienced occupational balance. Questionnaire results can be analyzed by considering each item separately or all together. Example items are: “When I think of a typical week, I have just enough to do” and “I have a balance between different occupations in my everyday life (employment, home and family chores, leisure occupations, rest and sleep)”. The OBQ was developed in Sweden and has good content validity, good internal consistency, and sufficient test-retest reliability [53].

##### Emotional intelligence

The Trait Emotional Intelligence Questionnaire - Short Form (TEIQue-SF) [35] is used to measure emotional intelligence. TEIQue-SF assesses the ability to emotionally handle different situations. It is an indicator of the ability to cope with task-induced stress. The TEIQue-SF has 30 items that can be responded to on a seven-point scale ranging from 1 (completely disagree) to 7 (completely agree). The following is an example of an item: “I can deal effectively with people”. The TEIQue-SF has been found to have good psychometric properties [35]. The TEIQue-SF was translated into Swedish in 2017 by Dåderman A., Grankvist G., and Ingelgård A., University West, Sweden, and Ronthy M., Amfora Future Dialogue and Hellström Å., Stockholm University, Sweden with permission from Prof. K. V. Petrides, Department of Psychology, University College London, UK. Back translation was done by Ramell International. Cross-cultural adaption has been done but not published yet [7].

##### Salutogenic health indicators

The Salutogenic Health Indicator Scale (SHIS) is associated with salutogenic and holistic descriptions of health. It has been developed with the support of theories related to the concepts of health and wellbeing. The SHIS has a varied and broad health perspective covering, cognitive, physical, and psychosomatic health. The SHIS are divided into two different dimensions of health indicators: intrapersonal characteristics and interactive functions. The SHIS employs a semantic differential – each question is answered on a six-point scale ranging from a positive to a negative wording of opposites. In this study, the SHIS has one overall question: “How have you been feeling over the past four weeks?” The response format is “Over the last four weeks I have been feeling...” with the options scoring 1 to 6 points. Here, 1 is negative (unhealthy) and 6 is positive (healthy). All items are estimated as one index. High scores indicate better health. The minimum and maximum scores are, respectively, 12 and 72 points. The SHIS has been developed and scientifically tested in connection with health promotion in the workplace life of two hospitals in Sweden, and its validity and reliability have been shown to be high [5].

##### Social interaction/teamwork

The General Nordic Questionnaire (QPS Nordic) is a general questionnaire for measuring psychological and social factors at work. It includes job and organization characteristics as well as individual, work-related attitudes. This study uses five questions about social interaction/teamwork. They measure how support from superiors, coworkers, friends, and relatives is experienced/perceived. Each question can be answered on a four-step ordinal scale ranging from “never” (scored at 0) to “often” (scored at 4). High scores indicate strong social interaction. The following is an example of a question related to social interaction: “If needed, can you get support and help with your work from your coworkers?” The questions in QPS Nordic are well tested in terms of reliability and validity [8, 21, 22]. For the assessments during the education programs, questions are selected and modified to fit a student perspective, e.g. substituting “classmates” and “studies” for “coworkers” and “work”.

##### Work experience

To measure experiences of work and workplace-related situations, the Work Experience Measurement Scale (WEMS) will be used. The instrument is based on the salutogenic theory. The content relates to operational factors at work. As the statements that must be responded to are framed positively, results can be interpreted from a salutogenic perspective. WEMS has 32 statements divided into six dimensions. These are: supportive work conditions (seven statements), e.g. “We encourage and support each other at work”; internal experience/perception of work (six statements), e.g. “I feel that my work is meaningful”; autonomy (four statements), e.g.: “I decide my own work pace”; time experience (three statements), e.g. “I do not need to work more than my scheduled hours”; management (six statements), e.g.: “My boss is available when I need him/her”; and, change processes (six statements), e.g.: “The change process was carried out via open dialogue”. Statement responses are on a six-point Likert scale ranging from 1 (“agree completely”) to 6 (“disagree completely”). This gives the minimum and maximum scores of, respectively, 32 and 192. High scores indicate a positive experience/perception of work. WEMS was developed among personnel working at hospitals in Sweden, and the WEMS scale has shown high validity and reliability [32, 34].

### Data analysis

This study is a survey of all students in the included programs at the collaborating universities. Thus, the sample size is based on the number of students starting their programs in 2018. Based on the number of students admitted in 2017, the total number to be invited was 2000. It was expected that at least 40% (*n* = 800) would respond to the initial questionnaire. No power analysis was made before deciding the number of participants. We received 852 usable surveys at baseline. Based on previous longitudinal studies, the initial drop-out from baseline can be expected to be around 20% and then 30% at subsequent follow-ups. However, since this study runs throughout the education programs, the expected drop-out rate is expected to be lower in step 1 (i.e. the second and third assessments during the programs). It is expected to be higher at the last follow-up (i.e. the assessments during early working life). The external drop-out/non-response rate will be analyzed to determine if there are any systematic discrepancies. These analyses will be based on available background data from the questionnaire and from each university registrar, and on available information on program completion rates (taking into account program switching, moves to other universities and other unspecified reasons).

Descriptive statistics and comparisons between groups will be used to describe tha basic feauters of tha data in the study (e.g. gender, sociodemographic factors, and education programs) and includes Chi2 tests; Mann-Whitney tests for non-parametric variables; and, independent sample t-tests for parametric variables. Paired tests and other appropriate statistical methods are to be used to study possible changes over time. The significance level is to be set at α = 0.05. Data will be analyzed statistically using SPSS Statistics, version 24 (IBM Corp, Armonk, New York, USA).

## Discussion

This study will target important research on sustainable working life in healthcare and social work professionals. With many such professionals currently leaving or considering leaving, their professions, sustainability is seen as a challenge. An active approach to handling this challenge as early as when students are on education programs is crucial. Consequently, this longitudinal multicenter study is designed to explore health-promoting factors in different higher education programs within healthcare and welfare. Using a range of validated tools (all of which target health promotion), the study will measure health-promoting factors. This approach contrasts with those of previous studies in the area. These have more often taken a pathogenic perspective (i.e. a risk factor approach).

It is hoped that the results will contribute to predicting possible salutogenic factors that, for students on programs in higher education, are important for working life sustainability, and that it will map out the possible development of salutogenic factors and processes over time. This study is performed by researchers working at six universities within the Swedish framework for “Health Research in Collaboration”. Thus, the results will be useful for increasing education quality by stimulating the implementation of new strategies to better prepare our students for a sustainable working life within healthcare and social work.

The strengths of this study are the longitudinal design and its use of several validated instruments focusing on health-promoting factors. All of this is being applied in a single study into approaches for sustainable work. As with other longitudinal studies, there is always a risk of drop-out over the study period. Thus, to find study-facilitating strategies, the number of participants at baseline, and participant drop-out calculations were discussed within the research team. Furthermore, all students starting one of the included programs at the six universities in 2018 were invited to participate in the study. No power analysis was made for this study. The use of power analysis for determining sample size is needed for, 1) calculating statistical analyses and, 2) for appropriate generalization to the population [38]. The current study is seen as a total population survey. However, sufficient statistical strength should be achieved to be able to draw conclusions and, the results will only be generalized for students at the included universities.

## Data Availability

The datasets and materials used and/or analyzed during the current study are available from the corresponding author on reasonable request.

## Abbreviations

GDPR: General Data Protection Regulation
IAF: Interactive Function
IPC: Intrapersonal characteristics
LANE: A longitudinal analysis of nursing education
OBQ: Occupational Balance Questionnaire
QPS Nordic: The General Nordic Questionnaire
SHIS: Salutogenic Health Indicator Scale
SOC: Sense of coherence
TEIQue-SF: Trait Emotional Intelligence Questionnaires – Short Form
WEMS: Work Experience Measurement Scale
WHO: World Health organization

## References

[1] AFA (2015). Psykiska diagnoser i kontaktyrken inom vård, skola och omsorg.

[2] Antonovsky A (1987). Unraveling the mystery of health: How people manage stress and stay well.

[3] Antonovsky A (1996). The salutogenic model as a theory to guide health promotion. Health Promot Int.

[4] Blake H, Harrison C (2013). Health behaviours and attitudes towards being role models. Br J Nurs.

[5] Bringsen A, Andersson HI, Ejlertsson G (2009). Development and quality analysis of the Salutogenic Health Indicator scale (SHIS). Scand J Public Health.

[6] Bringsen A, Andersson HI, Ejlertsson G, Troein M (2012). Exploring workplace related health resources from a salutogenic perspective. Results from a focus group study among healthcare workers in Sweden. Work.

[7] Dåderman Anna M., Ingelgård Anders, Koopmans Linda (2020). Cross-cultural adaptation, from Dutch to Swedish language, of the Individual Work Performance Questionnaire. Work.

[8] Dallner M, Lindström K, Elo A-L, Skogstad A, Gamberale F, Hottinen V (2000). Användarmanual för QPSNordic.

[9] Dooris M, Doherty S (2010). Healthy universities--time for action: a qualitative research study exploring the potential for a national programme. Health Promot Int.

[10] Eriksson M, Lindstrom B (2005). Validity of Antonovsky’s sense of coherence scale: a systematic review. J Epidemiol Community Health.

[11] Eriksson M, Lindstrom B (2006). Antonovsky’s sense of coherence scale and the relation with health: a systematic review. J Epidemiol Community Health.

[12] Frogeli E, Rudman A, Ljotsson B, Gustavsson P (2018). Preventing stress-related ill health among newly registered nurses by supporting engagement in proactive behaviors: development and feasibility testing of a behavior change intervention. Pilot Feasibility Stud.

[13] Håkansson C, Ahlborg G (2010). Perceptions of employment, domestic work, and leisure as predictors of health among women and men. J Occup Sci.

[14] Hakansson C, Bjorkelund C, Eklund M (2011). Associations between women's subjective perceptions of daily occupations and life satisfaction, and the role of perceived control. Aust Occup Ther J.

[15] Hasson D, Gustavsson P (2010). Declining sleep quality among nurses: a population-based four-year longitudinal study on the transition from nursing education to working life. PLoS One.

[16] Langius A, Bjorvell H (1993). Coping ability and functional status in a Swedish population sample. Scand J Caring Sci.

[17] Lindmark U, Wagman P, Wahlin C, Rolander B (2018). Workplace health in dental care - a salutogenic approach. Int J Dent Hyg.

[18] Lindstrom B, Eriksson M (2005). Salutogenesis. J Epidemiol Community Health.

[19] Lindstrom B, Eriksson M (2006). Contextualizing salutogenesis and Antonovsky in public health development. Health Promot Int.

[20] Lindström B, Eriksson M (2010). The hitchhiker’s guide to salutogenesis: Salutogenic pathways to health promotion.

[21] Lindström K, Borg V, Dallner M, Elo A-L, Gamberale F, Knardahl S (1995). Measurement of psychological and social factors at work:–description of selected questionnaire methods employed in four Nordic countries.

[22] Lindström K, Elo A-L, Skogstad A, Dallner M, Gamberale F, Hottinen V (1997). QPS Nordic. General Nordic questionnaire for psychological and social factors at work User’s Guide TemaNord, 603.

[23] Lindwall U (2018). Sjukfrånvaron på svensk arbetsmarknad. Sjukskrivningar längre än 14 dagar och avslut inom 180 dagar i olika branscher och yrken. Socialförsäkringsrapport 2018:2.

[24] Ljungblad C, Granström F, Dellve L, Åkerlind I (2014). Workplace health promotion and working conditions as determinants of employee health. Int J Workplace Health Manag.

[25] Lovgren M, Gustavsson P, Melin B, Rudman A (2014). Neck/shoulder and back pain in new graduate nurses: a growth mixture modeling analysis. Int J Nurs Stud.

[26] Malik S, Blake H, Batt M (2011). How healthy are our nurses? New and registered nurses compared. Br J Nurs.

[27] Mayer CH, Boness C (2011). Interventions to promoting sense of coherence and transcultural competences in educational contexts. Int Rev Psychiatry.

[28] Mayer CH, Krause C (2011). Promoting mental health and salutogenesis in transcultural organizational and work contexts. Int Rev Psychiatry.

[29] McCuaig L, Quennerstedt M, Macdonald D (2013). A salutogenic, strengths-based approach as a theory to guide HPE curriculum change. Asia Pac J Health Sport Phys Educ.

[30] Mittelmark M, Bull T (2013). The salutogenic model of health in health promotion research. Glob Health Promot.

[31] Mittelmark M, Sagy S, Eriksson M, Bauer G, Pelikan J, Lindström B, Espnes G (2017). The handbook of salutogenesis in.

[32] Nilsson P, Andersson HI, Ejlertsson G (2013). The work experience measurement scale (WEMS): a useful tool in workplace health promotion. Work.

[33] Nilsson P, Andersson IH, Ejlertsson G, Troein M (2012). Workplace health resources based on sense of coherence theory. Int J Workplace Health Manag.

[34] Nilsson P, Bringsen A, Andersson HI, Ejlertsson G (2010). Development and quality analysis of the work experience measurement scale (WEMS). Work.

[35] O'Connor P, Nguyen J, Anglim J (2017). Effectively coping with task stress: a study of the validity of the trait emotional intelligence questionnaire-short form (TEIQue-SF). J Pers Assess.

[36] Petrides K. V., Pita Ria, Kokkinaki Flora (2007). The location of trait emotional intelligence in personality factor space. British Journal of Psychology.

[37] Por J, Barriball L, Fitzpatrick J, Roberts J (2011). Emotional intelligence: its relationship to stress, coping, well-being and professional performance in nursing students. Nurse Educ Today.

[38] Price James H., Daek Joseph A., Murnan Judy, Dimmig Jaime, Akpanudo Sutoidem (2005). Power Analysis in Survey Research: Importance and Use for Health Educators. American Journal of Health Education.

[39] Rudman A, Gustavsson JP (2012). Burnout during nursing education predicts lower occupational preparedness and future clinical performance: a longitudinal study. Int J Nurs Stud.

[40] Rudman A, Gustavsson P, Hultell D (2014). A prospective study of nurses’ intentions to leave the profession during their first five years of practice in Sweden. Int J Nurs Stud.

[41] Rudman A, Omne-Ponten M, Wallin L, Gustavsson PJ (2010). Monitoring the newly qualified nurses in Sweden: the longitudinal analysis of nursing education (LANE) study. Hum Resour Health.

[42] Ruiz-Aranda D, Extremera N, Pineda-Galan C (2014). Emotional intelligence, life satisfaction and subjective happiness in female student health professionals: the mediating effect of perceived stress. J Psychiatr Ment Health Nurs.

[43] Severinsson E, Holm AL (2012). Knowledge gaps in nursing leadership - focusing on health care systems organisation. J Nurs Manag.

[44] Socialstyrelsen (2018). Bedömning av tillgång och efterfrågan på personal i hälso- och sjukvård och tandvård. Nationella planeringsstödet 2018.

[45] Stoller JK (2011). Teamwork, teambuilding and leadership in respiratory and health care. Can J Respir Ther.

[46] The public Health Agency of Sweden (2018). Public Health reporting. The national public health survey.

[47] Thune C. Standards and guidelines for quality assurance in the European higher education area: Report, European Association for Quality Assurance in the European Higher Education; 2005.

[48] Thylefors I, Persson O, Hellstrom D (2005). Team types, perceived efficiency and team climate in Swedish cross-professional teamwork. J Interprof Care.

[49] Utriainen K, Ala-Mursula L, Kyngas H (2015). Hospital nurses’ wellbeing at work: a theoretical model. J Nurs Manag.

[50] Wagman P (2012). Conceptualizing life balance from an empirical and occupational therapy perspective.

[51] Wagman P, Bjorklund A, Hakansson C, Jacobsson C, Falkmer T (2011). Perceptions of life balance among a working population in Sweden. Qual Health Res.

[52] Wagman P, Hakansson C (2014). Exploring occupational balance in adults in Sweden. Scand J Occup Ther.

[53] Wagman P, Hakansson C (2014). Introducing the occupational balance questionnaire (OBQ). Scand J Occup Ther.

[54] Whitehead D (2011). Before the cradle and beyond the grave: a lifespan/settings-based framework for health promotion. J Clin Nurs.

[55] WHO (2006). The world health report 2006: working together for health.

[56] World Medical Association (WMA) (2013). World medical association declaration of Helsinki: ethical principles for medical research involving human subjects. JAMA.

